# Mechanisms of Increase of Winter Wheat Frost Resistance Under Tebuconazole Treatment at Early Stage of Growth: Role of Hormone- and Reactive Oxygen Species-Mediated Signaling Pathways

**DOI:** 10.3390/plants14030314

**Published:** 2025-01-21

**Authors:** Anna V. Korsukova, Irina V. Lyubushkina, Natalya S. Zabanova, Ekaterina V. Berezhnaya, Elizaveta A. Polyakova, Tamara P. Pobezhimova, Kuzma A. Kirichenko, Nikolay V. Dorofeev, Lyubov V. Dudareva, Olga I. Grabelnych

**Affiliations:** Siberian Institute of Plant Physiology and Biochemistry, Siberian Branch of the Russian Academy of Sciences, Irkutsk 664033, Russia; avkorsukova@gmail.com (A.V.K.); ostrov1873@yandex.ru (I.V.L.); pavnatser@mail.ru (N.S.Z.); ekaterina809@mail.ru (E.V.B.); polyackova.elizaveta727@yandex.ru (E.A.P.); pobezhimova@sifibr.irk.ru (T.P.P.); gargamelle@yandex.ru (K.A.K.); nikolay.v.dorofeev@gmail.com (N.V.D.); laser@sifibr.irk.ru (L.V.D.)

**Keywords:** growth, cold hardening, frost resistance, fluridone, tebuconazole, water-soluble carbohydrates, reactive oxygen species, hormones, *Triticum aestivum* L.

## Abstract

1, 2, 4-triazole derivatives, including tebuconazole, have been reported to show positive physiological effects in cereals apart from fungicidal activity and to increase plants’ tolerance against temperature stress. This study investigates the mechanisms of increasing frost resistance of etiolated winter wheat (*Triticum aestivum* L., “Irkutskaya” variety) seedlings by tebuconazole-based seed dresser “Bunker” (1.5 μL g^−1^ of seeds) and tebuconazole (30 μg g^−1^ of seeds). To identify ABA-dependent and ABA-independent pathways of frost resistance, we used fluridone (FLD, 5 mg L^−1^), an inhibitor of endogenous abscisic acid (ABA) synthesis. FLD effectively inhibited the accumulation of carotenoids in the shoots and prevented the formation of carotenoids caused by the “Bunker” and tebuconazole. In non-hardened seedlings, FLD stimulated coleoptile and first leaf growth, but did not suppress the growth inhibitory effects of “Bunker” and tebuconazole. In shoots of hardened seedlings, FLD reduced the retarding effect of tebuconazole. Regardless of seedling age, temperature, and the protectant treatment, FLD had no effect on the sugar content in the shoots. FLD did not essentially influence frost resistance induced by “Bunker” and tebuconazole in cold-hardened seedlings. Fluridone increased H_2_O_2_ content and guaiacol peroxidase activity under control conditions (both with tebuconazole and without tebuconazole) and during cold hardening (in seedlings from seeds treated with tebuconazole). ABA levels in cold-hardened seedlings treated with FLD alone, tebuconazole alone, or a combination of the two were two to three times lower than in untreated hardened seedlings. Changes in indole-3-acetic and salicylic acids in response to FLD and tebuconazole treatment indicate complex interactions with signaling cellular systems. Our results suggest that tebuconazole activates ABA-independent pathways more strongly than ABA-dependent pathways in enhancing frost resistance. The potential mechanisms of tebuconazole action in plant cells are discussed.

## 1. Introduction

Low temperature is one of the important environmental factors that limits the growth, productivity, and distribution of plants around the world. An increase in the cold and frost resistance of plants to unfavorable low temperatures occurs as a result of cold hardening (cold acclimation)—a complex of structural and functional rearrangements, including changes in lipid and fatty acid compositions, synthesis of stress proteins, and accumulation of compounds with a cryoprotective function [1,2,3,4]. There is evidence that plant resistance to low temperatures can be increased by treatment with a variety of chemicals, including plant growth and development regulators and fungicides. Currently, systemic fungicides, which are inhibitors of C14 demethylation, are extensively used in agriculture. Triazole-based plant growth regulators are widely used in plants for growth, stress, and disease control [5]. Triazoles are characterized by low phytotoxicity, efficacy at small doses, environmental friendliness, and a wide spectrum of antifungal activity [6,7].

1, 2, 4-triazole derivatives with fungicidal properties, which are inhibitors of sterol and terpenoid synthesis, are the most widely used ones [7]. In addition, 1, 2, 4-triazole derivatives are retardants, since they inhibit internode elongation in cereal crops and the growth of plant axial organs by disrupting the biosynthesis of gibberellins (GB), namely gibberellic acid (GA3) [8,9]. Their other effects on the balance of phytohormones were also found. In particular, their ability to increase the content of abscisic acid (ABA) was shown [8,10,11,12,13,14]. GB and ABA biosynthesis is carried out from a common precursor—mevalonic acid (isoprenoid pathway). GBs are formed from acetate and mevalonate via ent-kaurene. GA12 synthesis (a common precursor of GB) from ent-kaurene is catalyzed by enzymes of the cytochrome P450 monooxygenase group. The enzyme activity is inhibited by 1, 2, 4-triazole derivatives, leading to the suppression of GB synthesis; mevalonic acid begins to be consumed for the synthesis of other phytohormones, in particular, ABA [9]. The mode of action of paclobutrazole, which is one of 1, 2, 4-triazole derivatives, has been well studied. Paclobutrazole was shown to inhibit the oxidation of ent-kaurene to ent-kaurenoic acid, thereby reducing GA production and increasing ABA content [10,12]. Paclobutrazole has a stress-protective effect and protects plants from abiotic stressors by maintaining the water ratio, cell membrane stability, and pro/antioxidant balance [12]. Triazole fungicides have been observed to play a role in the protection of the photosynthetic apparatus, an increase in photosynthetic pigments, soluble protein, and sugars, the effect on respiration, changes in the composition of fatty acids, as well as the enhancement of plant resistance to temperature stress, water deficiency, chloride salinization, and oxidative stress [9,12,15,16,17,18,19,20,21,22].

One of the representatives of 1, 2, 4-triazoles is tebuconazole (1-(4-chlorophenyl)-4,4-dimethyl-3-(1H-1,2,4-triazol-1-ylmethyl)-3-pentanol), which is contained in preparations widely used in agriculture. Tebuconazole is mainly used as a preventive and curative systemic fungicide, being effective against all types of cereal rust, septoria, smut, root rot, and seed mold. Like the other triazole derivatives, tebuconazole has, in addition to its fungicidal properties, a retardant effect by inhibiting GB biosynthesis [7,23]. In rapeseed cultivation, tebuconazole is used as a retardant to enhance branching and reduce overall plant height [24,25]. An increase in grain yield was one of the positive effects of treating wheat plants with tebuconazole [26].

According to Korsukova et al. [27,28] and Pobezhimova et al. [21,29], the tebuconazole-based seed protectant, “Bunker” (water suspension concentrate, tebuconazole content 60 g/L, JSC “Avgust” Inc., Russia) increases the resistance of etiolated seedlings of the cereals (spring wheat, winter wheat, and rye) to low temperatures. “Bunker” is systemic fungicide with both preventive and curative properties. It increases cold and frost resistance through its retardant effect and its impacts on carbohydrate, protein, fatty acid, and respiratory metabolism [27,28,29]. The “Bunker” effectively decontaminates the seeds [30]. A different effect of “Bunker” and pure tebuconazole was shown in winter wheat mitochondria [29]. Therefore, it is crucial to confirm that the increased frost resistance observed in wheat treated with tebuconazole-based seed dresser is specifically associated with the action of tebuconazole. It is known that triazole derivatives, including tebuconazole, increase the contents of ABA [8,10,12,14]. ABA is a polyfunctional plant hormone that plays an important role in mechanisms of increasing resistance to cold stress [31]. Increasing ABA synthesis might be the cause of high cold and frost resistance of cereals observed after treatment by a tebuconazole-based protectant.

To test the hypothesis regarding the involvement of endogenous ABA in the tebuconazole effect, we examined the influence of the endogenous ABA synthesis inhibitor (fluridone herbicide, FLD) on the physiological action of the tebuconazole-based protectant “Bunker” and pure tebuconazole in the early stages of growth of winter wheat (3-day-old etiolated seedlings). Fluridone inhibits synthesis and the accumulation of carotenoids and ABA biosynthesis [32]. The use of fluridone may reveal ABA-dependent and ABA-independent pathways of increasing frost resistance. Morpho-physiological (length and biomass of shoots and frost resistance of seedlings) and biochemical (content of carotenoids, sugars, hormones, hydrogen peroxide, lipid peroxidation, catalase, and peroxidase activity) analyses were carried out.

We showed that the fluridone action blocking the ABA synthesis weakened the signal transduction through the ABA-dependent pathway, reducing freezing tolerance. The protective effect of tebuconazole under freezing conditions was associated mainly with the activation of ABA-independent signal transduction.

## 2. Results

### 2.1. Carotenoids Content

**Figure 1:**
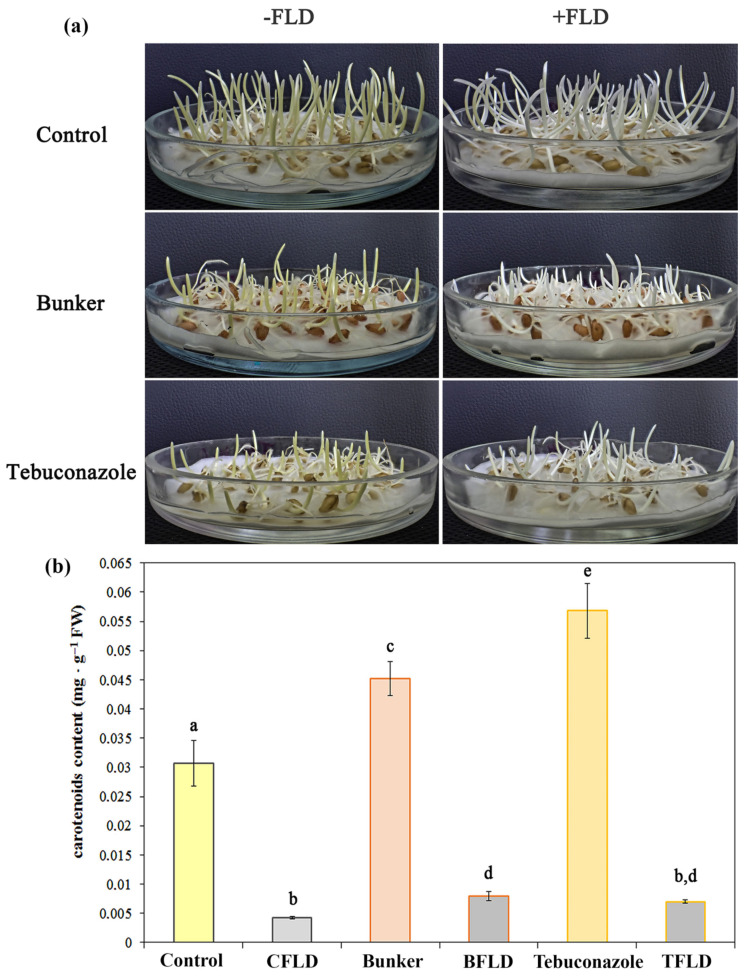
Influence of the fluridone, tebuconazole-based protectant “Bunker”, and tebuconazole on the content of carotenoids in shoots of 3-day-old etiolated winter wheat seedlings. (**a**) Photo of 3-day-old seedlings. (**b**) Quantitative content of carotenoid: control—plants grown from untreated seeds; CFLD—plants grown from untreated seeds in fluridone solution (5 mg·L^−1^); Bunker—plants grown from “Bunker”-treated seeds (1.5·μL g^−1^ of seeds); BFLD—plants grown from “Bunker”-treated seeds in fluridone solution; tebuconazole—plants grown from tebuconazole-treated seeds (30 μg·g^−1^ of seeds); and TFLD—plants grown from tebuconazole-treated seeds in fluridone solution. Data are presented as mean ± SD (*n* = 6). Different letters in the graph indicate the presence of statistically significant differences between the mean values of variants according to Fisher’s LSD method (*p* ≤ 0.05).

Fluridone is known to inhibit the synthesis of endogenous ABA by inhibiting the formation of carotenoids [32]. Figure 1a presents clearly seen differences in the synthesis of carotenoids in shoots of control (Control) and fluridone-treated seedlings (CFLD) of winter wheat, as well as differences in the shoots of seedlings grown from seeds treated with the tebuconazole-containing protectant (Bunker) and tebuconazole (Tebuconazole). Shoots grown from seeds treated with the “Bunker” and tebuconazole are bright yellow, while control fluridone-treated (CFLD), Bunker-fluridone-treated (BFLD), and tebuconazole-fluridone-treated (TFLD) shoots are colorless.

Fluridone was found to effectively reduce (by 87%) carotenoid content in the shoots of 3-day-old seedlings (CFLD) grown from untreated seeds (Figure 1b). Seed treatment with “Bunker” and tebuconazole led to the increase in the content of carotenoids in the shoots. The content was 1.4 and 1.8 times higher in BFLD and TFLD shoots, respectively, compared to control seedlings (Figure 1b). Fluridone almost completely blocked the “Bunker”- and tebuconazole-induced increase in carotenoids, reducing levels by 83% and 87% in BFLD and TFLD treatments, respectively (Figure 1b).

### 2.2. Growth Parameters Under Control and Cold Hardening

To assess the role of endogenous ABA in tebuconazole’s retardant effect, we studied how fluridone affected coleoptiles and first leaf length (Table 1) in winter wheat seedlings under different conditions. The study included both unhardened etiolated seedlings of various ages (3, 5, and 10 days) and hardened seedlings (+2 °C, 7 days), with seeds treated with either “Bunker” or tebuconazole alone.

“Bunker” showed a significant growth-inhibiting effect on seedlings (Table 1). The protectant had a more pronounced effect on the growth of coleoptiles, with inhibition ranging from 31 to 46% (Table 1). The strongest coleoptile growth inhibition (46%) was observed in 3-day-old seedlings and remained at this level in cold-hardened seedlings (Table 1). Unlike coleoptiles, first leaf growth (and consequently, overall shoot growth) was less sensitive to the tebuconazole-containing protectant. Shoot growth inhibition decreased with seedling age: 43% in 3-day-old seedlings, 25% in 5-day-old seedlings, and 12% in 10-day-old seedlings compared to the control (Table 1).

During cold hardening of 3-day-old seedlings, the growth-inhibiting effect of “Bunker” remained at approximately the same level as it was before hardening (about 45%). The growth-inhibiting effect of “Bunker” was associated with the tebuconazole effect (Table 1). Inhibition of the length of coleoptiles of non-hardened seedlings by pure tebuconazole was also most pronounced in 3-day-old seedlings and amounted to 51%, which was not significantly different from the inhibition caused by “Bunker”. Inhibition of the length of coleoptiles of 5- and 10-day-old seedlings was 43% and 46%, respectively. In cold-hardened seedlings, tebuconazole inhibited the growth of coleoptiles by 48%, which also did not differ significantly from inhibition with “Bunker” (Table 1).

In 3- and 5-day old seedlings, the growth inhibitory effect of tebuconazole on the length of shoots did not significantly differ from the effect of “Bunker” and amounted to 49% and 24%, respectively, while in 10-day-old seedlings it was 20%. In cold-hardened seedlings, the growth-inhibiting effect of tebuconazole was 43%, and did not significantly differ from the effect of “Bunker” (Table 1).

Treatment with fluridone led to a statistically significant increase in the length of coleoptiles and shoots both in unhardened seedlings of winter wheat of different ages (3, 5, and 10 days) and hardened (+2 °C, 7 days) ones grown from untreated (Control) and treated with “Bunker” and tebuconazole seeds (Table 1). The only exception is the lack of a stimulating effect of fluridone on the shoot length of hardened seedlings grown from seeds untreated with the protectant. Thus, treatment with fluridone led to the increase in the length of coleoptiles of winter wheat grown from untreated seeds by 9–18%, and the increase in the length of shoots by 5–20% (Table 1). The increase in coleoptile length under the action of fluridone in seedlings grown from seeds treated with “Bunker” was 11–14%, and the increase in shoot length was 14–23%. Fluridone led to an increase in the length of seedlings from seeds treated with tebuconazole, so the increase in the length of coleoptiles was 11–32%, and that of shoots was 21–52% (Table 1).

Shoot growth turned out to be more sensitive to fluridone. Under the action of fluridone, the length of shoots inhibited by the protectant reached the level of control seedlings (165 mm in BFLD, 151 mm in TFLD, and 156 mm in the Control) on the 10th day of growth (Table 1).

Significant inhibition of shoot length was observed in 3-day-old seedlings grown from seeds treated with “Bunker” and tebuconazole. This was accompanied by a decrease in the fresh and dry biomass of shoots (Table 2). Treatment with fluridone did not lead to changes in the fresh and dry biomass of shoots (coleoptile and first leaf) in control plants or in seedlings from seeds treated with “Bunker” (Table 2). Fluridone treatment resulted in a statistically significant increase in the fresh and dry weight of 3-day-old and cold-hardened plants from tebuconazole-treated seeds (Table 2).

### 2.3. Water-Soluble Carbohydrate Content in Shoots Under Control and Cold Hardening

The further studies examined changes in water-soluble carbohydrate content in shoots of etiolated winter wheat seedlings under fluridone treatment. This included seedlings grown from untreated (Control) and treated (“Bunker” and tebuconazole) seeds at three stages: 3-day-old, 10-day-old, and cold-hardened (7 days at 2 °C) seedlings.

**Figure 2:**
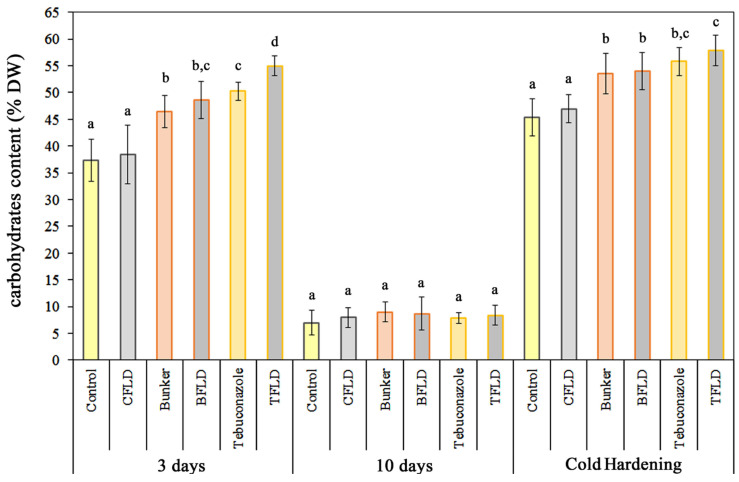
Influence of fluridone, tebuconazole-based protectant “Bunker”, and tebuconazole on the content of water-soluble carbohydrates in 3- and 10-day-old shoots of etiolated winter wheat seedlings and after cold hardening (2 °C, 7 days). Data are presented as mean ± SD (*n* = 3). Various lowercase letters in the graph indicate the presence of statistically significant differences between the mean values of variants within the plants of the same group (3 days, 10 days, and cold hardening) according to Fisher’s LSD method (*p* ≤ 0.05). Designations of variants coincide with designations in Figure 1.

As shown in Figure 2, treatment of winter wheat seeds with the tebuconazole-containing protectant “Bunker” and tebuconazole led to a statistically significant increase in water-soluble carbohydrate content in shoots of unhardened and hardened seedlings. By day 3 of growth, shoot water-soluble carbohydrate content increased by 28% and 35% under “Bunker” and tebuconazole treatment, respectively, compared to control; during cold hardening, these increases were 21% and 35%. Fluridone treatment did not significantly affect water-soluble carbohydrate content in any experimental variants, except in seedlings from tebuconazole-treated seeds (TFLD) (Figure 2).

### 2.4. Frost Resistance of Seedlings

**Figure 3:**
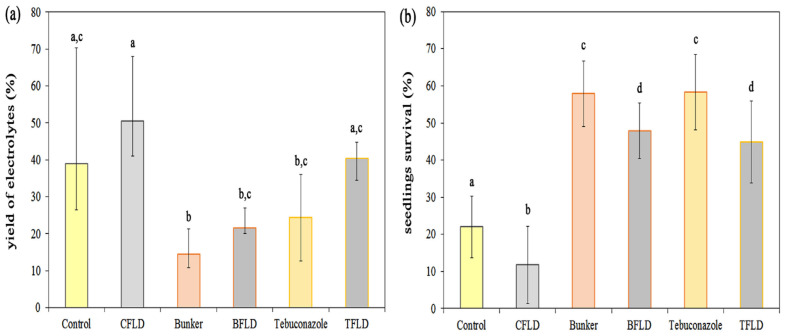
Influence of fluridone, tebuconazole-based protectant “Bunker”, and tebuconazole on release of electrolytes and survival of cold-hardened (2 °C, 7 days) etiolated winter wheat seedlings after exposure to negative temperature (–8 °C, 24 h). (**a**) Release of electrolytes from shoot tissues, Me [25%; 75%] (*n* = 10). (**b**) Survival of seedlings after 5 days of recovery, M ± S.D (*n* = 8). Different letters in the graph indicate the presence of statistically significant differences between the values of variants according to the Kruskal–Wallis one-way analysis of variance on ranks and all pairwise multiple comparison procedures, Student–Newman–Keuls method (for (**a**)) and Fisher’s LSD (for (**b**)) (*p* ≤ 0.05). Designations of variants coincide with designations in Figure 1.

Korsukova et al. [27,28] showed that the tebuconazole-containing seed dresser “Bunker” increased the frost resistance of cereal seedlings. It was necessary to find out whether ABA was involved in increasing frost resistance. In order to do that, we studied the effect of fluridone on the permeability of cell membranes in shoot tissues and the survival rate of cold-hardened winter wheat seedlings (2 °C, 7 days) grown from seeds treated with “Bunker” and tebuconazole after exposure to a damaging temperature (–8 °C, 24 h). Cell membrane permeability was assessed by measuring the release of electrolytes immediately after subzero temperature exposure (Figure 3a), while seedling survival rates were evaluated 5 days after freezing during their growth under control conditions (Figure 3b).

In shoots of seedlings grown from seeds treated with “Bunker” and tebuconazole, the release of electrolytes after freezing was observed to be noticeably lower (by 47% and 37%, respectively) than in the control (Figure 3a). In seedlings grown from untreated seeds (CFLD), fluridone treatment increased electrolyte leakage from shoot tissues by 53% after freezing. Electrolyte leakage from shoot tissues after freezing increased by 48% in seedlings grown from seeds treated with both the “Bunker” and fluridone (BFLD), and by 65% in seedlings treated with both tebuconazole and fluridone (TFLD). In seeds treated with “Bunker”, the release of electrolytes from shoot tissues was significantly lower than in control seedlings, whereas in seeds treated with tebuconazole, the yield of electrolytes did not significantly differ compared to the control group (Figure 3a).

Changes in the release of electrolytes from shoot tissues immediately after exposure to negative temperatures correlated with the ability of fluridone, tebuconazole-based protectant “Bunker”, and tebuconazole to modulate the survival rate of cold-hardened winter wheat seedlings after their growth under control conditions (Figure 3b). An increase in the survival rate of seedlings from seeds treated with “Bunker” and tebuconazole and a decrease in the survival rate of fluridone-treated seedlings from untreated (CFLD), “Bunker”-treated (BFLD), and tebuconazole-treated (TFLD) seeds were observed (Figure 3b).

To identify the roles of hormone and reactive oxygen species (ROS) signaling pathways, we analyzed the biochemical parameters following fluridone and tebuconazole treatments.

### 2.5. Hydrogen Peroxide Content, Lipid Peroxidation, and Antioxidant Enzyme Activity Under Control and Cold Hardening

**Figure 4:**
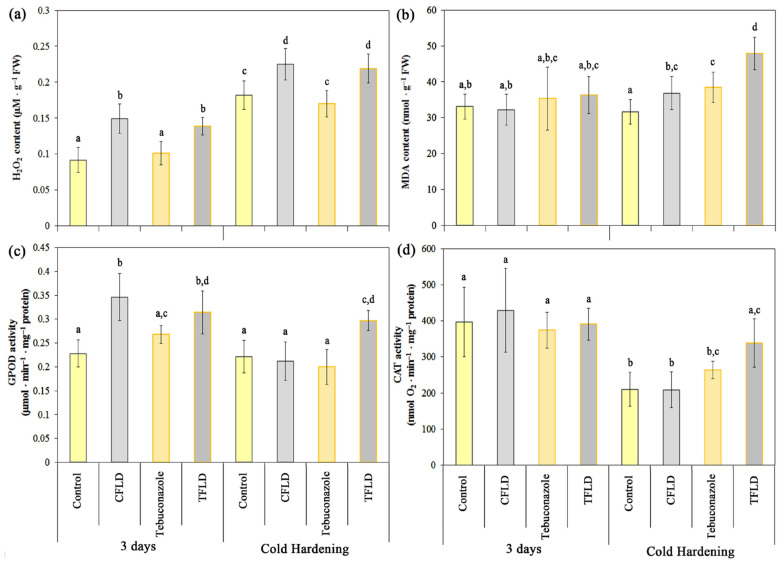
Influence of fluridone and tebuconazole on the hydrogen peroxide (**a**) and malondialdehyde (MDA) content (**b**), guaiacol peroxidase (GPOD) (**c**) and catalase (CAT) (**d**) activities in shoots of 3-day-old and cold-hardened (2 °C, 7 days) etiolated winter wheat seedlings. Data are presented as mean ± SD (*n* = 3). Different letters in the graph indicate the presence of statistically significant differences between the mean values of variants according to Fisher’s LSD method (*p* ≤ 0.05). Designations of variants coincide with designations in Figure 1.

Hydrogen peroxide is one of the main ROS involved in the development of oxidative stress. Previous studies have shown that triazoles regulate reactive oxygen species (ROS) content and lipid peroxidation (LPO) in plant cells under stress conditions, including low temperature stress [22]. There is also information stating that fluridone acts as a pro-oxidant [33]. In this regard, we assessed the content of hydrogen peroxide (Figure 4a) and lipid peroxidation activity, measured as malondialdehyde (MDA) content resulting from the reaction with thiobarbituric acid (TBA) (Figure 4b) in shoots of 3-day-old and cold-hardened winter wheat seedlings grown from untreated and tebuconazole-treated seeds in water, as well as in fluridone solution. We also studied the activity of catalase (CAT) (Figure 4c) and guaiacol peroxidase (GPOD) (Figure 4d) involved in the detoxification of hydrogen peroxide.

The cold hardening of seedlings resulted in a statistically significant increase in H_2_O_2_ content in all variants compared to 3-day-old (non-hardened) seedlings (Figure 4a). At the same time, the hydrogen peroxide content in plants grown from untreated (Control) and tebuconazole-treated seeds did not differ statistically significantly in either 3-day-old or hardened seedlings. Thus, seed treatment with tebuconazole does not result in the generation of H_2_O_2_ either in 3-day-old seedlings or during cold hardening, while the hydrogen peroxide content remains at the level of that in control plants (Control). At the same time, growing seedlings in fluridone solution (CFLD and TFLD) led to a statistically significant increase in H_2_O_2_ levels in plants grown from untreated (Control) and tebuconazole-treated seeds, both 3-day-old and cold hardened. Thus, the hydrogen peroxide content in 3-day-old seedlings from untreated (CFLD) and tebuconazole-treated (TFLD) seeds grown in fluridone solution was 39% and 27% higher, respectively, than in 3-day-old seedlings from untreated (Control) and tebuconazole-treated seeds grown in water (Figure 4a).

The data we obtained on the content of the LPO product—MDA (Figure 4b)—indicate that an increase in hydrogen peroxide does not cause oxidative stress either in 3-day-old seedlings (in all experimental variants) or in cold-hardened seedlings, with the exception of the TFLD variant. Thus, in cold-hardened seedlings from tebuconazole-treated seeds grown in fluridone solution (TFLD), we observe a statistically significant increase in MDA of 34% compared to the cold-hardened control variant (Control) (Figure 4b).

It was found that in 3-day-old plants, an increase in GPOD activity occurs only in seedlings grown in fluridone solution, both in plants from untreated (CFLD) and tebuconazole-treated (TFLD) seeds, by 34% and 15%, respectively (Figure 4c). In cold-hardened plants, a statistically significant increase in GPOD activity was observed in seedlings from tebuconazole-treated seeds grown in fluridone solution (TFLD). With other treatments, GPOD activity remained at the level of that in 3-day-old unhardened plants (Control, 3 days) (Figure 4c).

The study of CAT activity (Figure 4d) showed that in 3-day-old seedlings, no treatment increased the activity of this enzyme. In cold-hardened plants, a statistically significant decrease in CAT activity was observed compared to 3-day-old plants, dropping by 40–45%. Only in the TFLD (cold-hardened seedlings from seeds treated with tebuconazole and grown in fluridone solution), did CAT activity not significantly differ from the control group (3-day-old seedlings).

### 2.6. Endogenous Hormone Content Under Control and Cold Hardening

**Figure 5:**
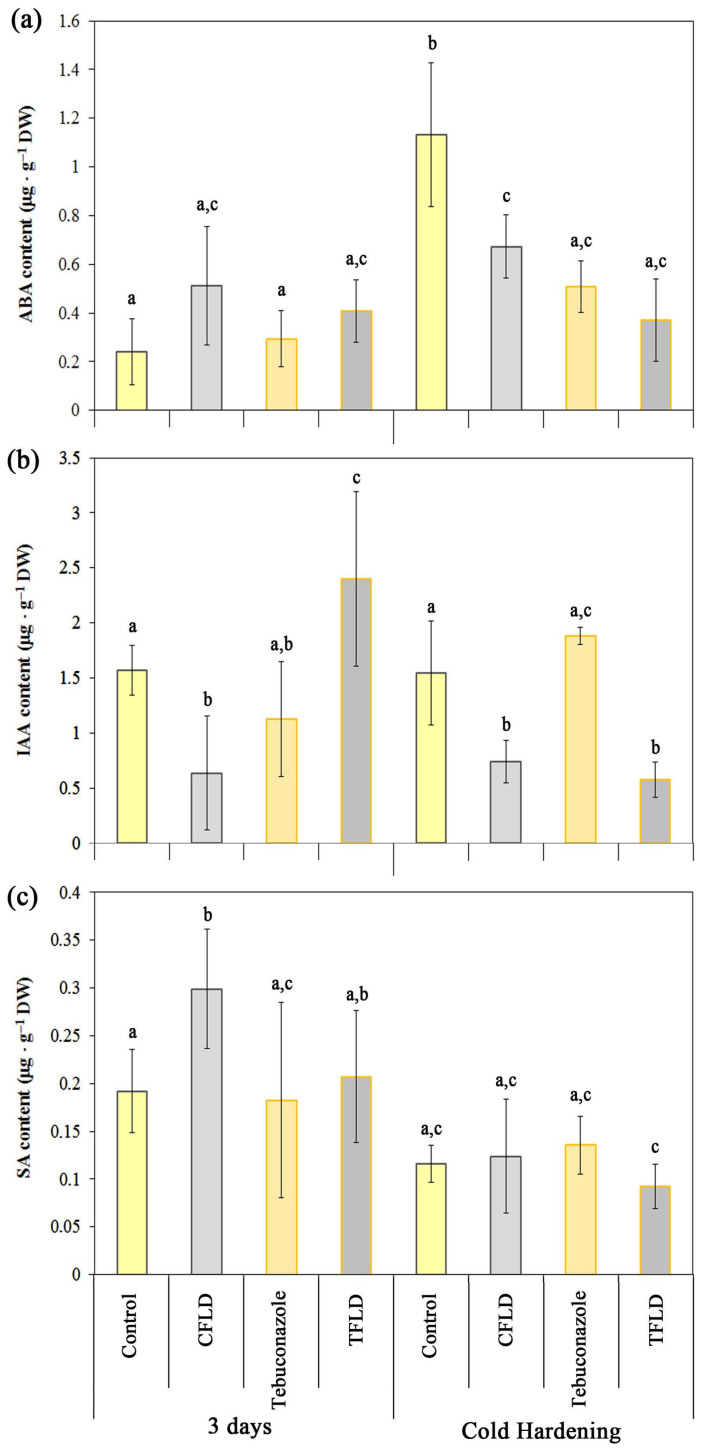
Influence of fluridone and tebuconazole on the abscisic acid (ABA) (**a**), indole-3-acetic acid (IAA) (**b**), and salicylic acid (SA) (**c**) in shoots of 3-day-old and cold-hardened (2 °C, 7 days) etiolated winter wheat seedlings. Data are presented as mean ± SD (*n* = 3). Different letters in the graph indicate the presence of statistically significant differences between the mean values of variants according to Fisher’s LSD method (*p* ≤ 0.05). Designations of variants coincide with designations in Figure 1.

When studying the effect of fluridone, tebuconazole, and their combined treatment on the hormonal status of etiolated control and hardened winter wheat seedlings, we initially focused on the change in the ABA content. On the one hand, this was because there is sufficient information in the literature on the direct and indirect effect of these substances on the ABA level in plants. Therefore, we needed to find out whether such an effect occurred in our work. On the other hand, ABA is probably the key hormone regulating the process of low-temperature hardening in plants; therefore, it is essential to thoroughly study any alterations in its concentration. It has been demonstrated that exposure of etiolated seedlings to low positive temperatures (2 °C) for a period of 7 days resulted in an almost 6-fold increase of in the content of ABA in shoot tissues (Figure 5a).

Fluridone, tebuconazole, and their combination did not affect ABA levels in the tissues of control seedlings (Figure 5a). However, in hardened seedlings, treatment with fluridone, tebuconazole, or both reduced ABA content by two to three times (Figure 5a). Thus, our study revealed significant differences in the effects of the tested substances on ABA content in etiolated winter wheat seedlings, depending on the temperature. In this regard, the following question arises: could these differences be due to the indirect effect of fluridone or tebuconazole on other components of the hormonal regulation? To answer this question, we studied changes in the concentration of active IAA in winter wheat seedlings.

The study of IAA content in the shoots of etiolated seedlings revealed a high basic level of this hormone, which remained unchanged during low-temperature hardening (Figure 5b). It was found that fluridone treatment reduced IAA content in the tissues of both control and hardened seedlings by almost three times (Figure 5b). Tebuconazole, in turn, did not significantly change IAA levels either under control conditions or during low-temperature hardening. Of particular interest was the combined effect of tebuconazole and fluridone. In control seedlings, tebuconazole-fluridone treatment increased IAA content by about 1.5 times, while in hardened seedlings, there was a decrease in IAA content of more than 3 times (Figure 5b). This finding suggests that each of the studied substances most likely has an indirect rather than direct effect on IAA synthesis, possibly through other components of the hormonal network.

Another important hormone involved in seed germination, growth regulation, and plant response to various stress factors is SA. Its basic levels vary in different plants [34]. In our study, SA contents in both control and hardened winter wheat seedlings were quite low (Figure 5c). Although there were no statistically significant changes in SA, the hardened seedlings showed a tendency towards a decrease in the level of this hormone. The effects of fluridone, tebuconazole, and their combination on SA content varied depending on the temperature conditions. In control seedlings, fluridone increased SA levels by approximately 1.5-fold, whereas in hardened seedlings, it had no effect on SA content (Figure 5c). Tebuconazole alone did not have a significant effect on SA content, either under control conditions or during low-temperature hardening. However, when combined with fluridone, it prevented an increase in SA levels in control seedlings and contributed to a decrease in SA in hardened seedlings (Figure 5c). Thus, the data obtained on the changes in SA levels in response to fluridone and tebuconazole treatments also indicate the complex effect of these substances on signaling cellular systems.

## 3. Discussion

Enhanced ABA biosynthesis and subsequent accumulation are among the general plant responses to stress [35]. Thus, during cold hardening, the plant hormonal balance shifts towards elevated concentrations of growth inhibitors, mainly ABA, which causes growth suppression [36]. The retardant effect of triazoles is also associated with their influence on the balance of phytohormones [8,10,12,13,23]. It is known that triazole compounds affect the isoprenoid pathway, which is responsible for the synthesis of essential plant hormones including GA, ABA, and cytokinins [37]. Triazoles affect GA biosynthesis by inhibiting the oxidation of ent-kaurene to ent-kaurenoic acid [8,9]. It can be assumed that the disruption of GA synthesis should lead to increased ABA production. Indeed, in a number of studies, it has been stated that triazole compounds enhanced cytokinin and ABA levels while reducing GA content [38].

The tebuconazole-containing seed dresser “Bunker” (60 g L^−1^ of tebuconazole) increased cold and frost resistance in etiolated cereal seedlings (winter wheat and rye, spring wheat) by inhibiting shoot growth, increasing sugar content, increasing unsaturation of fatty acids in membrane lipids, inducing the synthesis of stress proteins such as dehydrins, and reducing respiratory metabolism [27,28,29]. The growth-inhibiting effects of this tebuconazole-containing protectant persisted when winter wheat plants were grown under light conditions [39]. It remained to be determined to what extent these effects were specifically attributable to tebuconazole itself. It was unclear whether the identified effects were ABA dependent or ABA independent.

Being a 1, 2, 4-triazole derivative, tebuconazole presumably acts at the level of the cytochrome P450 monooxygenase enzyme, which catalyzes the synthesis of GA_12_ (a common precursor of other GBs) from ent-kaurene [23]. Inhibition of this enzyme leads to the suppression of GB synthesis and the switch of the metabolic isoprenoid pathway to ABA synthesis from mevalonic acid (a common precursor for GB and ABA synthesis) [9]. Suppressed GB synthesis and increased ABA contents result in the inhibition of the growth of plant axial organs [40]. ABA, the main stress hormone in plants, triggers the synthesis of stress proteins, including ABA-dependent dehydrins [1]. In addition, ABA stimulates the accumulation of water-soluble carbohydrates [41], which in turn can enhance dehydrin synthesis [42]. An increase in ABA can lead to an increase in the activity of desaturases, resulting in higher concentrations of polyunsaturated fatty acids [43]. ABA was shown to be involved in the stabilization of cell membranes in the cold [44]. Tebuconazole also suppresses the cytochrome respiratory pathway at the level of complex I of the mitochondrial respiratory chain; a decrease in respiration intensity saves substrates (water-soluble carbohydrates), which also favors accumulation [29]. Electron transport through the alternative pathway via alternative oxidase (AOX) is not inhibited. At low temperatures, it can be important for the prevention of oxidative stress in mitochondria.

Growth inhibition, sugar accumulation, increased fatty acid unsaturation in cell membrane lipids, and dehydrin synthesis all enhance plant survivability at low temperatures. For ABA-dependent pathways of increasing cold and frost resistance, ABA is involved in the process. To verify the assumptions made, it was necessary to test the dependence of the revealed effects of the tebuconazole-containing protectant on ABA synthesis. During cold acclimation of plants, ABA-independent pathways are also induced [31]. Blocking ABA synthesis with targeted inhibitors may help to reliably determine its role in triazole-mediated effects. Fluridone, a herbicide inhibiting endogenous ABA synthesis, was used in the present research. Fluridone was confirmed to be effective in inhibiting ABA synthesis in various plants including wheat [32,45,46].

The effect of “Bunker”, as well as that of pure tebuconazole, was more pronounced in coleoptiles at an early seedling growth phase (on the 3rd day) and persisted during cold hardening of 3-day-old seedlings (Table 1). Inhibition of shoot growth (coleoptile and leaves) for seedlings from seeds treated with “Bunker” and tebuconazole was also more pronounced on day 3 of seedling growth and persisted during cold hardening, but diminished under control growth conditions (Table 1). Fluridone stimulated the growth of coleoptiles and shoots in seedlings from both untreated seeds and those treated by “Bunker” and tebuconazole. Thus, the ABA synthesis inhibitor partially reversed the growth-retarding effects of “Bunker” and tebuconazole. Similar data on wheat coleoptile growth stimulated by fluridone were obtained by other researchers [47]. When studying the effect of fluridone on the growth of coleoptiles of different types of cereals, they noted that fluridone treatment enhanced coleoptile growth by 14% in wheat and 67% in rice, compared to the control groups.

Notably, fluridone exhibited no stimulatory effect on the shoots of hardened seedlings grown from seeds not treated with “Bunker”. Conversely, fluridone reduced the retardant effect of “Bunker” and tebuconazole. Based on previous reports of triazoles increasing ABA synthesis [8,10,12] and our experimental results showing changes in coleoptile and shoot length under fluridone treatment (Table 1), we conclude that the retarding effect of tebuconazole and tebuconazole-containing “Bunker” is largely independent of ABA under control conditions and is likely related to GB biosynthesis inhibition. However, during cold hardening in organs still growing (first leaf), ABA has a more noticeable effect on growth and, possibly, on resistance.

The effect of triazole retardants on leaf photosynthesis is well documented. Triazole derivatives, including tebuconazole, are known to increase carotenoid and chlorophyll contents [48]. The use of pure tebuconazole and the mixture of chlorocholine chloride with tebuconazole in winter rape during the spring growing season increased the content of photosynthetic pigments in leaves [25]. In turn, fluridone effectively inhibits the formation of carotenoids, which are precursors of ABA biosynthesis [32]. The data we obtained confirm that fluridone suppresses carotenoid synthesis in the shoots of seedlings grown from untreated seeds and prevents carotenoid formation caused by treatment with “Bunker” and tebuconazole (Figure 1). Inhibition of carotenoid synthesis in shoots by treatment with fluridone proves the appropriateness of its use to inhibit endogenous ABA synthesis.

One of the consequences of seedling growth inhibition after being subjected to triazole derivatives is a more economical consumption of sugars, which was demonstrated by Korsukova et al. [28] in winter wheat and rye by using the tebuconazole-containing protectant “Bunker”. Tebuconazole treatment resulted in a rise in sugar yield in sugar beet under drought stress conditions [20]. Water-soluble carbohydrates fulfill several functions in plant cells during low-temperature hardening due to their osmoregulatory, antifreezing, cryoprotective, and antioxidant properties. In addition, water-soluble carbohydrates are the main substrates for respiration. ABA is evidenced to affect the metabolism of water-soluble carbohydrates and the activity of enzymes involved in water-soluble carbohydrate accumulation during hardening [41]. However, the results obtained with fluridone did not reveal the dependence of the elevated sugar contents on ABA synthesis in the shoots of winter wheat seedlings treated with the tebuconazole-containing protectant and tebuconazole (Figure 2). Thus, it can be assumed that the increase in the sugar content in cereals grown from seeds treated with “Bunker” and tebuconazole is not associated with ABA. Previous research demonstrated that “Bunker” effectively inhibited both phosphorylating and non-phosphorylating respiration in winter wheat mitochondria during malate oxidation [29]. This treatment also decreased the respiratory control coefficient by affecting the function of respiratory chain complex I. Inhibition of the substrate oxidation rate by mitochondria, induced by the tebuconazole-containing protectant and tebuconazole, can be one of the reasons for an increase in water-soluble carbohydrates in the shoots of unhardened and hardened cereal seedlings (Figure 2).

Reduced growth and increased sugar content are some of the factors that increase plant resistance to low temperatures [1,41]. The tebuconazole-containing protectant increases cold and frost resistance in cereal seedlings, causing the following physiological effects: reduced growth and accumulation of sugars, induction of dehydrin synthesis, and increased fatty acid unsaturation in membrane lipids, etc. [27,28]. In seedlings grown from “Bunker”-treated seeds, there was a decrease in the release of electrolytes and an increase in the survival rate compared to the control (Figure 3). The results obtained are consistent with the data on the ability of triazoles (tebuconazole and paclobutrazole) to reduce the release of electrolytes [12,26]. The survival rates of cold-hardened seedlings from seeds treated with “Bunker” and pure tebuconazole after freezing did not differ significantly (Figure 3b). However, seed plants treated with “Bunker” exhibited a reduced electrolyte yield, which was significantly different compared to the control (Figure 3a). Thus, it can be assumed that “Bunker” and pure tebuconazole have different effects on the permeability of cell membranes. This discrepancy could be attributed to the composition of Bunker, which contains additional components beyond the active ingredient (tebuconazole). Similarly, differences in composition might explain the varied effects of Bunker and pure tebuconazole on the respiratory activity of winter wheat mitochondria [29].

Shaki et al. [49] concluded that there is a positive and strong correlation between triazole treatment and the activity of antioxidant systems in plants under stress. Presumably, triazoles are able to induce stress-like symptoms to stimulate the antioxidant system. This may be the reason for the overall increase in H_2_O_2_ levels in plants [49]. However, our experiments only showed a trend towards an increase in H_2_O_2_ in seedlings from tebuconazole-treated seeds (Figure 4a). In contrast, fluridone led to an increase in H_2_O_2_ in shoots (Figure 4a). It should be noted that under the same treatment (TFLD), cold-hardened seedlings showed a decrease in the content of carotenoids (Figure 1b), playing a protective role against oxidative stress. The reduction in carotenoids combined with increased electrolyte release (Figure 3a) and decreased survival rates (Figure 3b) suggests that fluridone can act as a pro-oxidant. This finding aligns with previous research [33].

According to our results, the increase in frost resistance under the influence of the tebuconazole-containing protectant “Bunker” occurs with the participation of mechanisms unrelated to ABA, although ABA-dependent mechanisms certainly play an important role in the mechanisms of frost resistance development. We did not observe an ABA increase in control or in hardened winter wheat seedlings treated with tebuconazole (Figure 5a). On the contrary, in cold-hardened seedlings, ABA concentrations even decreased in response to tebuconazole treatment, whereas in control seedlings, this treatment had no visible effect (Figure 5a). Considering the reasons for such changes, it is important to remember that the endogenous hormone content is determined by the balance of its synthesis, decay, and transport, as well as conjugation with amino acids and sugars and conversion into inactive forms.

Based on our results, we can conclude that ABA synthesis was minimal in 3-day-old etiolated winter wheat seedlings during active growth (Figure 5a). This was indirectly supported by high levels of IAA, which acts as an ABA antagonist and functions downstream of ABA, particularly during seed germination [50]. It should be noted that high IAA levels are typical for seedlings grown in the dark [50]. Similar results were obtained by Kosova et al. working with two cultivars of spring wheat when, against the background of high IAA contents under control conditions, very low ABA concentrations were detected [2]. In our work, the absence of active ABA synthesis in control winter wheat seedlings was also evidenced by the fact that the level of this hormone did not change when seedlings were treated with fluridone, an inhibitor of ABA and carotenoid synthesis (Figure 5a) [32]. The fluridone concentration used in the work was sufficient, as indicated by a significant decrease in carotenoids during this treatment (Figure 1b). Moreover, the inhibitory effect of fluridone was so pronounced that it was not removed by the action of tebuconazole when they were applied together (Figure 1b). It can be assumed that some amount of ABA in etiolated winter wheat shoots was preserved, due to its conversion from an inactive form (e.g., from glucosyl ester) to an active one, or due to a decrease in its degradation rate [51]. Thus, it is logical that in the absence of ABA synthesis, the effect of tebuconazole on the content of this hormone was absent. At the same time, the effect of tebuconazole on the isoprenoid pathway was undisputed. This can be judged, in particular, by an increase in carotenoid content in the shoots of etiolated winter wheat seedlings when treated with tebuconazole. As carotenoids are a product of the isoprenoid pathway in plants and precursors of ABA, the increase in their content indicates the intensification of synthetic reactions of other branches of this process due to the blocking of GA synthesis [37,50]. Since ABA synthesis did not occur, carotenoids were not consumed, and their content in the tissues of seedlings increased (Figure 1b). It should also be noted that tebuconazole treatment caused inhibition of shoot growth of control seedlings (Table 1). Since no changes in IAA levels were observed in this case, it is likely that this inhibition was due to the effect of tebuconazole on other growth hormones, namely GA (Figure 5b).

ABA is known to be essential in plant responses to cold stress. A number of works have shown intensification of endogenous ABA biosynthesis under cold stress or improvement of cold and frost stress resistance in plants after application of exogenous ABA [52]. Indeed, in our work, we observed a rise in ABA levels in winter wheat seedlings after cold hardening (Figure 5a). Moreover, the results of our experiments with fluridone confirmed the important role of ABA in the formation of winter wheat frost resistance. Fluridone decreased the survival of seedlings when exposed to negative temperatures, which was accompanied by a decrease in ABA levels in shoots (Figure 5a). Contrary to expectations, tebuconazole did not increase ABA levels in hardened seedlings, while their survival rates under negative temperatures increased (Figure 5a). Moreover, tebuconazole prevented a fluridone-induced decrease in winter wheat seedling resistance to negative temperatures, but ABA contents were also reduced compared to ABA levels in hardened seedlings with no treatment (Figure 3b and Figure 5a). The increase in frost resistance in this case was probably due to ABA-independent signal transduction, realized, as is known today, through DREB1/CBF [53,54]. The protective effect of tebuconazole in our experimental setting might be attributed to its capacity to impede GA synthesis. For instance, the GA1 mutant of Arabidopsis with impaired GA production demonstrated higher freezing tolerance than wild-type plants [55]. At least partially, this effect is connected with GA-regulated GATA transcription factors (TFs) GNC and GNL, promoting the CBF1, CBF2, COR15A, and COR15B responsible for the frost resistance [55,56].

Analysis of changes in the active IAA and ABA levels in the tissues of winter wheat seedlings treated with fluridone and tebuconazole allows us to assume that their effect on the hormonal system of plants was multifaceted, involving various signaling pathways. Thus, despite the impact of tebuconazole on the isoprenoid pathway in plants, our study did not reveal evidence supporting its direct effect on ABA synthesis. Evidence suggests that the ABA level can be reduced, rather than elevated, under the influence of triazole compounds. This finding aligns with those of Zheng et al., who documented a decline in the content of GA, ABA, and IAA in the inflorescences of *Astragalus sinicus* L. when using paclobutrazole, another representative of the triazole group [57]. A stable decrease in the ABA content in winter wheat treated with fluridone under low-temperature treatment was a consequence of its direct effect on the synthesis of this hormone, but a decrease in the level of free IAA was precisely a consequence of its indirect effect. In the work of Stetsenko et al. on the effect of fluridone on the hormone content in crystal grass under NaCl-salinity conditions, a decrease in free IAA and an increase in IAA conjugates were shown in both control and NaCl stress conditions [33].

One of the potential mechanisms of this influence involves changes in activity and/or in expression of genes of GH3 family enzymes, which regulate homeostasis of hormones including IAA, jasmonic acid (JA), and SA. Amidosynthetases belonging to this family catalyze reactions of the conjugation of hormones with amino acids, converting them from active free form to inactive storage and transport form [51]. At the same time, changes in enzyme activity and gene expression of amidosynthetases affect the content of the entire hormone complex rather than individual hormones alone. Thus, it was shown that OsGH3-13-activated rice enhanced drought tolerance accompanied by reduced endogenous free IAA and ABA synthesis [58]. In our study, the IAA content decreased in control winter wheat seedlings treated with tebuconazole and both in control and hardened seedlings treated with fluridone, although these compounds are not known to have a direct effect on auxin synthesis (Figure 5b). The alterations in IAA levels observed in both control and hardened seedlings could be attributed to the impact of fluridone and tebuconazole on GH3. Due to the modulation of different signaling pathways, enzyme activity can both increase and decrease (Figure 5b). It is interesting that GH3.5 can act as a modulator not only in auxin signaling, but also in the SA-regulated system [59]. Our study demonstrated simultaneous changes in IAA and SA contents in winter wheat control seedlings under fluridone treatment and in hardened seedlings under combined treatment (Figure 5a,b). It is worthy of further investigation to determine whether the GH3 family is indeed involved in hormonal homeostasis in the context of fluridone and triazole treatments.

The canonical TIR1/AFB-Aux/IAA signaling pathway is an important component of auxin signaling, which might be involved in the regulation of energy metabolism, as well as hormonal homeostasis and cross-talk between signaling pathways [60]. It has been established that auxin binding to the receptor proteins TIR1 (Transport Inhibitor Response 1) and AFB (Auxin Signaling F-Box) causes ubiquitination and proteasomal degradation of proteins that are repressors of Aux/IAA transcription. Due to the cleavage of these proteins, ARF transcription factors are released, activating the corresponding genes [61]. It has been shown that auxin-dependent degradation of Aux/IAA is carried out by enzymes of the E3 ligase family, called SCFTIR1/AFB1–5. In this case, the implementation of auxin signal perception during substrate recognition requires direct binding of auxin to the F-box protein [62]. The process of identifying genes under the control of ARF is still ongoing, but among the identified genes of great interest are the aforementioned GH3 family genes, which encode amidotransferase enzymes responsible for conjugating not only IAA, but also JA with amino acids [63]. The GH3 family can potentially link the auxin signal transduction pathway with the activity of mitochondrial energy-dissipating systems, since JA is known to be involved in the regulation of AOX gene expression [64,65]. The fact that the functional activity of mitochondria can be regulated by auxin signaling pathways is evidenced by the results of studies with Arabidopsis mutants (rao3/big, rao4/pin-formed1, and rao5/multidrug-resistance1/abcb19), when the impairment of polar auxin transport resulted in an increase in AOX gene expression [66]. It was also shown that treatment of plants with the mitochondrial respiratory chain complex III inhibitor antimycin A blocked auxin signaling and induced the expression of the AOX1a gene [66]. Thus, the disruption of auxin homeostasis can lead to serious disturbances in cellular metabolism under the influence of unfavorable environmental factors. Our results suggest that reduced levels of active IAA following fluridone treatment may contribute to the decreased survival of seedlings exposed to freezing temperatures (Figure 3b and Figure 5b).

As mentioned above, SA has been shown to participate in plant defense responses to abiotic and biotic stress factors [67,68]. Several studies have stated that increased SA production was associated with reduced auxin biosynthesis and transport [39,40,69]. Interdependence between IAA and SA may be mediated by shared transcription factors and protein kinase CK2 regulating both SA and auxin biosynthetic pathways. Supporting this connection, we observed that when control winter wheat seedlings were treated with fluridone, IAA levels decreased while SA content increased (Figure 5b,c).

**Figure 6:**
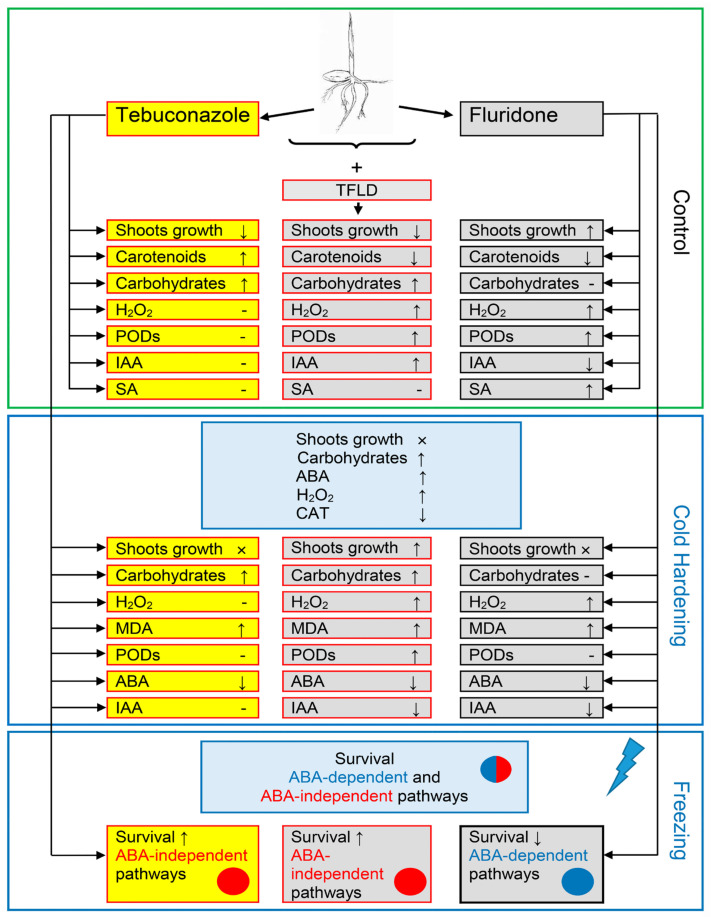
The generalized scheme of the influence of tebuconazole, fluridone, and its combined action (TFLD) on the increase in winter wheat frost resistance. Separate blocks show the influence of tebuconazole and FLD in optimal (Control, 24 °C), cold hardening (2 °C, 7 days), and freezing (−8 °C, 24 h) temperature conditions. ↑—parameter increase, ↓—parameter decrease, ×—growth stop, -—no parameter changes.

It is known that SA signaling, like other hormonal signaling pathways, is not a simple linear pathway. SA interacts with several compounds, including other hormones, polyamines, ROS, and several others [34]. Therefore, it has been stated that SA directly binds to and inhibits CAT2, thereby promoting H_2_O_2_ levels [69]. In our work, there were no changes in catalase activity when SA increased in control seedlings treated with fluridone (Figure 4d and Figure 5b). However, an increase in H_2_O_2_ levels was accompanied by an increase in SA levels (Figure 4a). It can be supposed that, in this case, SA contributed the ROS increase by inducing the succinate–quinone reductase activity (SDH, complex II in electron transport chain) in mitochondria [70]. SDH catalyzes the oxidation of succinate to fumarate and the reduction in ubiquinone to ubiquinol, and is a significant source of mitochondrial ROS production [70,71]. So, it can be assumed that the indirect effect of SA on the active IAA content was realized via the rise in mitochondrial ROS production. This was followed by H_2_O_2_ sulfenylation of Tryptofan Synthetase subunit 1 (TSB1), which is responsible for IAA synthesis, resulting in the inhibition of its activity [34]. Although SA can enhance antioxidant enzymes activities [72], indeed, when recording an increase in SA, we observed a parallel increase in guaiacol peroxidase activity in control seedlings treated with fluridone (Figure 4b and Figure 5c). However, these changes did not prevent ROS increase. In addition, fluridone significantly reduced the synthesis of carotenoids, which are also antioxidants. Consequently, one can propose that the negative influence of fluridone on the winter wheat frost resistance was attributed to its pro-oxidant action, which is facilitated through various pathways, probably including SA, antioxidant enzymes, non-enzymatic antioxidants, and mitochondria. The opposite effect of tebuconazole was largely associated with the activation of the ABA-independent signaling pathway during cold hardening. This treatment, however, could not completely exclude the negative effect of fluridone, as indicated by an increase in ROS and a decrease in carotenoids and IAA. The generalized scheme of the influence of tebuconazole and fluridone on frost resistance in winter wheat and the involvement of sugars, hormones, hydrogen peroxide and LPO products (MDA), catalase, and peroxidases are shown in Figure 6.

## 4. Materials and Methods

### 4.1. Plant Material, Fungicide and Inhibitor Treatments, and Growth Conditions

Etiolated winter wheat seedlings (*Triticum aestivum* L., ‘Irkutskaya’ variety) were used for the research. Winter wheat seeds were washed and disinfected with 0.1% potassium permanganate solution.

Some of the seeds were treated with an aqueous solution of the tebuconazole-based seed dresser (water-suspension concentrate) “Bunker” (1.5 μL g^−1^ of seeds) as detailed by Korsukova et al. [27]. The remaining seeds were treated with an aqueous solution of the tebuconazole (Tebuconazole PESTANAL^®^, Sigma-Aldrich, St. Louis, MO, USA) (water-suspension concentrate) (30 μg g^−1^ of seeds). For seed treatment, tebuconazole was dissolved in dimethyl sulfoxide (DMSO, Sigma-Aldrich, St. Louis, MO, USA).

**Figure 7:**
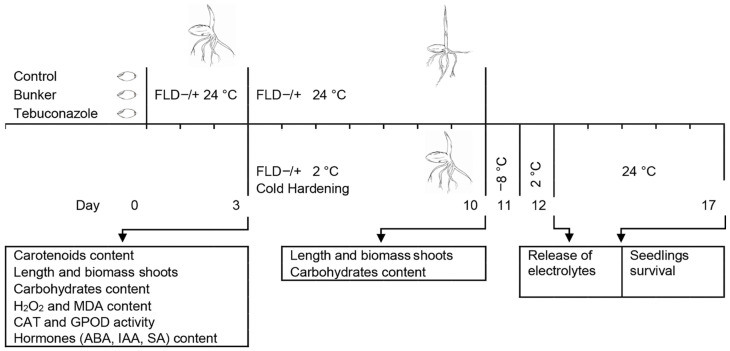
Scheme of study of effects of fluridone and tebuconazole on frost resistance in winter wheat seedlings. Control seeds (not treated at all), seeds treated with the tebuconazole-based protectant “Bunker” (1.5 μL·g^−1^ of seeds), and those treated with tebuconazole (30 μg·g^−1^ of seeds) were germinated in water or fluridone solution (FLD−/+) (5 mg·L^−1^) for 3 days at 24 °C. Subsequently, they were kept at 2 °C for 7 days (Cold Hardening) or left to grow at 24 °C (Control). Cold-hardened seedlings were frozen at −8 °C for 24 h, and then thawed at 2 °C for 24 h, and after 5 days of recovery at 24 °C, their survival rate was determined. The arrows indicate sampling time for morpho-physiological and biochemical analyses.

Untreated seeds, seeds treated with Bunker, and those treated with tebuconazole were germinated on wet filter paper in water (FLD-) or FLD solution (FLD+) (5 mg L^−1^) (Fluridon PESTANAL^®^, Sigma-Aldrich, St. Louis, MO, USA) in the dark at 24 °C in the incubator MIR-154 (Sanyo, Osaka, Japan) for up to 3, 5, and 10 days of age. FLD is a herbicide that slows down the synthesis of endogenous ABA by inhibiting the formation of carotenoids [45], and 0.1% DMSO solution was used for its preparation. The scheme of study of fluridone and tebuconazole influence on frost resistance in winter wheat seedlings is shown in Figure 7.

### 4.2. Temperature Treatments and Determination of Frost Resistance of Seedlings

Three-day-old etiolated seedlings from untreated seeds, Bunker-treated seeds, and tebuconazole-treated seeds were cold hardened for 7 days at 2 °C in a MIR-154 incubator (Sanyo, Japan).

To evaluate the growth parameters of unhardened and hardened winter wheat seedlings, the coleoptile and shoot (first leaf) length, as well as fresh (FW) and dry (DW) shoot biomasses of 30 seedlings were measured. The degree of growth inhibition was determined as the ratio of the difference in coleoptile (or shoot) length of plants grown from untreated seeds and that of plants grown from treated seeds to the coleoptile length of plants grown from untreated seeds. Dry weight was measured after drying shoots of seedlings in an oven heated at 72 °C for 48 h.

To determine frost resistance, cold-hardened etiolated seedlings were frozen at −8 °C for 24 h in the incubator MIR-154 (Sanyo, Japan). The seedlings were then thawed at 2 °C for a day. After that, some of the seedlings were used to determine the release of electrolytes (V, %), and some of the seedlings were left to recover and grow in the dark at 24 °C for 5 days. The number of seedlings that survived after the freezing was expressed as a percentage of their total number. Evaluation of the release of electrolytes from wheat shoot tissues was carried out using the conductivity meter HI 8734 (Hanna Instruments Inc., Nusfalau, Romania) according to the formula V = 100 × (Lt/Lk), where Lt is the electrical conductivity of the sample after the freezing and subsequent incubation for 24 h at room temperature and Lk is the electrical conductivity of the same sample after a 15 min boil [73]. 0.1 g of shoots in 50 mL of distilled water was used for the analysis.

### 4.3. Determination of Carotenoids Content

Carotenoid contents in shoots (coleoptile and first leaf) were estimated according to Gavrilenko and Zhigalova [74]. The samples of shoots (50 mg) were homogenized with a mortar and pestle and extracted in 80% chilled acetone (1 mL) with the addition of MgCO_3_. The homogenate was transferred into the test tubes (with the mortar and pestle being washed with 3 mL of acetone), left at 4 °C for 24 h, and then centrifuged at 7000× *g* for 10 min at 4 °C. The extract was poured into a 10 mL measuring tube. The pellet was mixed with a new portion of chilled 80% acetone (1 mL) and centrifuged again. The procedure was repeated several times until the pellet was completely discolored. Each time, the extracts were combined and the extract volume was adjusted to 7 mL with the solvent. Carotenoid content in the extract (C_car_, mg·L^−1^) was determined spectrophotometrically at a wavelength of 440 nm and calculated using the Wettstein’s formula C_car_ = 4695 × D_440_ − 0268 × C_a+b_. Pigment content was calculated using the formula F = C × V/(P × 1000), where C is pigment concentration (mg·L^−1^), V is volume of extract (mL), and P is plant fresh weight (g). Pigment content was expressed as mg·g^−1^ FW.

### 4.4. Determination of Water-Soluble Carbohydrate Content

The content of water-soluble carbohydrates in shoots was determined with 0.2% anthrone (Sigma-Aldrich, St. Louis, MO, USA) in concentrated sulfuric acid according, to Lyubushkina et al. [75], with some changes. The plant material was dried to a constant weight at 72 °C and ground in a mortar to powder. About 6–7 mg of dry ground material was transferred into test tubes, which were filled with boiling bidistilled water (10 mL). The tubes containing the extract were kept for a day in a thermostatic chamber at 24 °C. The supernatant was diluted 1:4 with bidistilled water, a 0.5 mL aliquot of the supernatant was taken, and double volume of anthrone solution in concentrated sulfuric acid was added (1 mL). After 15 min, the absorbance was measured at 620 nm. The content of water-soluble carbohydrates was calculated using a calibration curve based on sucrose, and expressed as a percentage (%) per g DW.

### 4.5. Determination of Hydrogen Peroxide and Lipid Peroxidation

Hydrogen peroxide content and lipid peroxidation were determined in extracts prepared from fresh powdered shoot tissue with 2.5 mL of 0.1% (*w*/*v*) trichloroacetic acid (TCA) per 0.5 g of tissue powder. The samples were previously frozen in liquid nitrogen and stored at −80 °C. The homogenate was centrifuged at 12,000× *g* for 15 min at 4 °C and supernatants were used for assays.

Hydrogen peroxide content was determined with xylenol orange [76]. The supernatant was diluted with 0.1% TCA in a ratio of 1:4 and a mixture of supernatant and reagent was prepared in a ratio of 1:1 (0.5 mL of each). The reagent composition was as follows: 0.5 mM FeSO_4_ × (NH_4_)_2_SO_4_ × 6H_2_O, 0.5% H_2_SO_4_, 200 μM xylenol orange (AppliChem, Germany) and 200 mM sorbitol (Gerbu, Heidelberg, Germany). The solution was shaken, incubated at room conditions for 30 min, and absorbance of the reaction mixture was determined at 560 nm. The H_2_O_2_ content was expressed as µM of H_2_O_2_ per g FW based on the H_2_O_2_ calibration curve (ranged from 0.02 to 10 µM).

Lipid peroxidation was estimated using the thiobarbituric acid (TBA) method, based on production of the MDA [77]. To determine the TBA-reactive products content, 0.5 mL of supernatant and 1 mL of 0.5% (*w*/*v*) TBA (Dia-M, Moscow, Russia) in 20% TCA were mixed and incubated in a boiling water bath for 30 min and the reaction was stopped by cooling the samples in ice. The samples were then centrifuged for 5 min at 12,000× *g* (MiniSpin, Eppendorf, Hamburg, Germany) and absorbance of the supernatant at 532 and 600 nm was measured (SmartSpec Plus, BioRad, Hercules, CA, USA). The accumulated MDA was calculated using the extinction coefficient TBA 155 mM^−1^·cm^−1^ after subtracting the nonspecific absorbance measured at 600 nm and expressed as nmols per gram of FW.

### 4.6. Determination of the Catalase and Guaiacol Peroxidase Activity

To measure the activity of catalase (CAT) and guaiacol peroxidase (GPOD) enzymes, fresh shoot samples (0.25 g) were frozen in liquid nitrogen and homogenized with 2.5 mL of 0.1 M K, Na-phosphate buffer (pH 7.8). After centrifugation at 15,000× *g* for 20 min, the resulting supernatant was used for the enzyme analyses. The supernatant was stored at 4 °C on ice in glass tubes.

The GPOD activity was determined by the increase in the optical density of the reaction medium due to guaiacol oxidation [78]. GPOD activity was measured in 0.1 M K, Na-phosphate buffer (pH 5.0), containing 10 mM hydrogen peroxide, 8 mM guaiacol (Sigma-Aldrich, St. Louis, MO, USA), and 0.05–0.1 mg protein at 470 nm for 3 min at 25 °C, using the extinction coefficient of 26.6 mM^−1^·cm^−1^. GPOD activity was expressed as the rate of tetraguaiacol formation per 1 mg protein of the sample: μmol·min^−1^·mg^−1^ protein.

Catalase activity was measured polarographically, by the rate of oxygen evolution formed during the decomposition of H_2_O_2_ [79] using a Clark type oxygen electrode (Oxytherm system, Hansatech Inst., Pentney, UK). In a polarographic cell (volume 1.4 mL) at 25 °C with 0.1 M K, Na-phosphate buffer (pH 7.8) extract (0.1–0.2 mg protein) was added, then hydrogen peroxide to a final concentration of 0.003%. Catalase activity was expressed in nmoles of oxygen released per minute per 1 mg of protein: nmol O_2_·min^−1^·mg^−1^ protein.

Protein concentration was determined using the Lowry method [80].

### 4.7. Determination of the Content of Endogenous Hormones

Hormone extraction and identification were carried out according to the method developed by Rudikovskii et al. [81]. The samples of shoots (1 g) were homogenized in liquid nitrogen. The internal standard was 4-methoxybenzoic acid. Extraction of phytohormones was carried out at 4 °C for 10 min in an ultrasonic bath, in 80% methanol with the addition of sodium diethylthiocarbamate as an antioxidant. Then, the samples were centrifuged (AllegraTM 64R centrifuge, Beckman Coulter, Brea, CA, USA) at 4 °C and 20,000× *g* for 20 min. The supernatant was removed and acidified with formic acid. The samples were purified using Sep-Pack C18 solid-phase extraction cartridges (Waters Corporation, Milfrod, MA, USA). Methanol was evaporated using an IR-1LT rotary evaporator (Labtex, Moscow, Russia) at 30 °C. The sample volume was adjusted to 5 mL with deionized water, acidified again with formic acid, and applied to an Oasis MAX cartridge (Waters Corporation, Milford, MA, USA). The cartridge was sequentially washed with 5% NH_4_OH and 100% methanol. The sample was then washed with 2% formic acid dissolved in methanol. Then, the sample was evaporated to dryness using a rotary evaporator. Trimethylsilyl derivatives of phytohormones were obtained by heating the samples at 70 °C for 30 min with N,O-bis-trimethylsilylacetamide and hexamethyldisilazane in a SHC-40-02 SPU drying cabinet (Smolensk SKTB SPU, Smolensk, Russia). Indole-3-acetic, abscisic, and salicylic acid preparations (Sigma Aldrich, St. Louis, MO, USA) were used as hormone standards. Endogenous hormones were analyzed by gas chromatography using a 5973N/6890N MSD/DS chromatograph mass spectrometer (Agilent Technologies, Santa Clara, CA, USA). The analysis was performed using a quadrupole mass spectrometer with electron impact (EI) ionization at 70 eV in total ion current recording mode. HP-5MS capillary columns (30 m × 250 μm × 0.50 μm) were used to separate endogenous hormones (TMS derivatives of phytohormones). The stationary phase was phenyl-methyl-polyxylosan. The mobile phase was helium, gas flow rate 1 mL·min^−1^. The operating temperatures were as follows: evaporator 250 °C, ion source 230 °C, detector 150 °C, and transfer line (connecting the chromatograph to the mass spectrometer) 280 °C. The scanning range was 41–450 a.m.u. The volume of the injected sample was 1 μL, the flow split was 5:1. Chromatography was performed in isocratic mode at 200 °C. Quantitative analysis of hormone content was performed using calibration curves constructed according to the corresponding standards. The NIST 05, Christie, and WILEY7 mass spectra libraries were used for the detection of plant hormones, as well as the comparison of the retention time with the retention times of standard compounds. The characteristic ions of TMS derivatives were: 4-metoxybenzoic acid (the internal standard)—267, 223, 193, 282, and 73; IAA—202, 319, 203, and 304; ABA—190, 183, 134, and 162; and SA—268, 267, 193, and 147.

### 4.8. Statistical Analyses

Statistical data processing was performed using SigmaPlot v. 14.0. In the figure captions, ‘n’ indicates the number of independent replicates. Data are presented as arithmetic mean (M) and standard deviation (±S.D.), or as median (Me) and interquartile range [25%; 75%]. Normality of distribution was checked using the Shapiro–Wilk test. Providing the distribution was normal, one-way analysis of variance and the procedure of multiple comparisons of means according to the Fisher LSD method were used to prove the presence of significant differences between the means. Providing the distribution was non-normal, the Kruskal–Wallis one-way analysis of variance on ranks and all pairwise multiple comparison procedures (Student–Newman–Keuls method) were used to prove the presence of significant differences. Differences between experimental data were considered statistically significant at *p* ≤ 0.05.

## 5. Conclusions

Our research showed that fluridone, at concentrations that inhibit the synthesis of carotenoids and consequent ABA biosynthesis, partially reduced the growth-inhibiting effect of the tebuconazole-based “Bunker” seed dresser and tebuconazole in coleoptiles and the first leaf of unhardened seedlings. In addition, fluridone reduced the retardant effect of the protectant and tebuconazole on the first leaf in cold-hardened seedlings. Fluridone caused electrolyte leakage and reduced the survival rate of seedlings grown from untreated seeds to a greater extent than that of seedlings grown from “Bunker”-treated and tebuconazole-treated seeds. Thus, we can conclude that the survival of etiolated winter wheat seedlings under freezing conditions was ensured by gene activation through ABA-dependent and ABA-independent signaling pathways. Fluridone, which blocks ABA synthesis, weakened signal transduction through the ABA-dependent pathway, thereby reducing freezing tolerance. Additionally, fluridone’s negative effect on the formation of cold and frost resistance mechanisms likely occurred through disruption of auxin homeostasis, specifically via SA and ROS, which blocked IAA synthesis and accelerated free IAA oxidation. A stable decrease in active IAA was observed with all fluridone applications and resulted in a decrease in signaling through the main auxin pathway TIR1/AFB-Aux/IAA, which controls the expression of many genes. Thus, fluridone can affect the homeostasis of other hormones, such as jasmonic acid, and through them, potentially, the energy metabolism of the plant cell, which is a key physiological process that determines the effectiveness of the stress response.

The protective effect of tebuconazole, both as a single treatment and in combination with fluridone, was mainly associated with the activation of ABA-independent signal transduction at low temperatures, which significantly increased the survival of winter wheat seedlings under freezing conditions. At the same time, partial compensation for the negative effect of fluridone on auxin homeostasis by tebuconazole, even under control conditions, was sufficient to stabilize plant metabolism, and prepared it for the action of stress environmental factors. The mechanisms of IAA metabolism and signaling remain to be fully elucidated and require further investigation. Such studies will not only provide new insights into how triazoles and fluridone affect plants, but will also enhance our understanding of auxin signaling pathways through TIR1/AFB-Aux/IAA and help identify new genes regulated by ARF.

## Figures and Tables

**Table 1:** Influence of fluridone, tebuconazole-based protectant “Bunker”, and tebuconazole on the length of coleoptiles and shoots of 3-, 5-, and 10-day-old etiolated winter wheat seedlings and after cold hardening (2 °C, 7 days). Data are presented as median (Me) and interquartile range [25%; 75%] (*n* = 3–4). The values following the same letters do not statistically differ significantly in plants of the same group (3 days, 5 days, 10 days, and cold hardening). Kruskal–Wallis one-way analysis of variance on ranks and all pairwise multiple comparison procedures (Student–Newman–Keuls method), *p* ≤ 0.05. Designations of variants coincide with designations in Figure 1.

Variants	Growth Period, Days			Cold Hardening
	3	5	10	
Coleoptile, mm				
Control	39 [35; 43] c	49 [46; 53] d	46 [43; 48] d	42 [38; 45] c
CFLD	45 [42; 47] d	58 [54; 62] e	50 [46; 53] e	46 [41; 49] d
Bunker	21 [18; 24] a	34 [31; 37] a, b	27 [25; 29] a, b	22 [20; 25] a
BFLD	24 [22; 27] b	38 [36; 43] a	30 [28; 33] c	25 [24; 29] b
Tebuconazole	19 [13; 22] a	28 [26; 30] c	25 [23; 27] a	22 [17; 24] a
TFLD	27 [23; 29] b	31 [29; 33] b, c	30 [25; 32] b, c	29 [26; 31] b
Shoot, mm				
Control	37 [31; 40] c	83 [75; 95] b	156 [131; 178] a	40 [34; 45] c
CFLD	39 [36; 43] d	93 [85; 104] c	182 [145; 211] b	36 [30; 41] a
Bunker	21 [18; 24] a	62 [58; 67] a	138 [121; 149] c	22 [20; 26] b
BFLD	25 [22; 27] b	71 [65; 78] d	165 [148; 181] a, b	27 [24; 30] d
Tebuconazole	19 [13; 22] a	63 [52; 71] a	125 [111; 137] d	23 [17; 27] b
TFLD	27 [23; 29] b	79 [69; 85] e	151 [136; 165] a	35 [32; 37] a

**Table 2:** Influence of fluridone, tebuconazole-based protectant “Bunker”, and tebuconazole on the fresh and dry biomasses of shoots of 3- and 10-day-old etiolated winter wheat seedlings and after cold hardening (2 °C, 7 days). Data are presented as mean ± SD (*n* = 3). Fresh and dry biomasses contain the weight of 30 seedlings. Values followed by different letters are statistically significantly different (*p* ≤ 0.05). One-way ANOVA followed by Fisher’s LSD multiple comparisons within plants of the same group (3 days, 10 days, and cold hardening). Designations of variants coincide with designations in Figure 1.

Variant	Growth Period, Days		Cold Hardening
	3	10	
Fresh Weight, g			
Control	1.047 ± 0.034 a	1.624 ± 0.667 a	1.042 ± 0.025 a
CFLD	1.016 ± 0.046 a	1.591 ± 0.624 a	0.965 ± 0.138 a
Bunker	0.573 ± 0.114 b	1.721 ± 0.359 a	0.638 ± 0.229 a
BFLD	0.633 ± 0.098 b	1.734 ± 0.507 a	0.788 ± 0.280 a
Tebuconazole	0.480 ± 0.142 c	2.760 ± 0.020 b	0.616 ± 0.149 b
TFLD	0.823 ± 0.033 d	2.462 ± 0.393 b	0.998 ± 0.211 c
Dry Weight, g			
Control	0.0871 ± 0.0036 a	0.1490 ± 0.0588 a	0.1200 ± 0.0115 a
CFLD	0.0944 ± 0.0078 a	0.1510 ± 0.0610 a	0.1250 ± 0.0281 a
Bunker	0.0591 ± 0.0094 b	0.1710 ± 0.0354 a	0.0889 ± 0.0361 a
BFLD	0.0667 ± 0.0069 b	0.1680 ± 0.0517 a	0.1080 ± 0.0437 a
Tebuconazole	0.0555 ± 0.0111 c	0.2750 ± 0.0059 b	0.0879 ± 0.0139 b
TFLD	0.0842 ± 0.0036 d	0.2500 ± 0.0412 b	0.1260 ± 0.0268 c

## Data Availability

The original contributions presented in the study are included in the article. Further inquiries can be directed to the corresponding author.
